# A Comprehensive Review of Schizophrenia and Antipsychotic Metabolism as a Predictor of Treatment Response

**DOI:** 10.7759/cureus.65279

**Published:** 2024-07-24

**Authors:** Mia Pham, Aydin Caglayan

**Affiliations:** 1 General Internal Medicine, St. George's Hospital, London, GBR; 2 General Surgery, Medway NHS Foundation Trust, London, GBR

**Keywords:** schizophrenia, treatment-resistant schizophrenia, pharmacogenetics, clozapine, cyp450 pathway

## Abstract

Some patients with schizophrenia fail to respond to standard antipsychotics and are considered treatment-resistant. In these cases, clozapine is the only antipsychotic with proven efficacy, but its use is complicated by severe adverse effects, complex monitoring requirements, and non-response.

Variation within the CYP450 enzymes CYP1A2, CYP2D6, CYP3A4, and CYP2C19 has been linked to the differential metabolism of antipsychotics. Testing for CYP450 single nucleotide polymorphisms may be a useful predictor of treatment resistance and could inform pharmacogenetic recommendations to identify potential treatment non-responders. Nonetheless, it remains uncertain whether differential antipsychotic metabolism is directly related to treatment efficacy.

This comprehensive narrative review endeavours to delve into the molecular and genetic basis of schizophrenia, and discuss the current treatments available. In particular, we aim to examine the aetiology of treatment resistance in schizophrenia through available literature and discuss current challenges within the field.

## Introduction and background

Overview of schizophrenia

Schizophrenia is defined as a chronic, relapsing mental health condition associated with considerable disability affecting all aspects of daily life, including social, educational, and occupational functioning [1,2]. This is due to alterations in a person’s perception, mood, thoughts, and behaviour.

Clinical presentation and incidence

Incidence

Schizophrenia is predicted to affect approximately 1% of the population worldwide and is ranked by the World Health Organization (WHO) within the top 10 diseases contributing to the global disability burden [3]. The mean incidence of schizophrenia is estimated to be one in 300 people worldwide, and it confers a two- to three-fold increased risk of all-cause mortality and a 20-fold increased risk of suicide [4,5]. Incidence has prominent variation according to factors such as sex, migrant status, urbanicity, economic status, and latitude [6].

Sex

There is a sex difference in schizophrenia risk, with incidence rate ratios of men to women being 1.42:1 [7].

Migrant Status

There are higher rates of schizophrenia within various migrant groups identified within a Danish population-based cohort study, with a relative risk of developing schizophrenia of 2.45 among first-generation immigrants [8].

Urbanicity

Incidence is observed to increase within urban settings when compared with rural regions [9]. Another Danish population-based cohort study by Vassos et al. found that birth in capital or large cities has an incidence rate ratio of 1.69 (p < 0.0001) for schizophrenia, and 1.36 (p < 0.0001) for schizoaffective disorders compared to those born in a rural area [10].

Economic Status

Socioeconomic deprivation is associated with an increased incidence of schizophrenia [11]. Nevertheless, one needs to consider potential confounders such as drug use and crime.

Latitude

There is a positive correlation between latitude and schizophrenia incidence, especially in males, according to an ecological study carried out in 2006 [12]. In addition, numerous studies have demonstrated an excess of winter/spring births in relation to the incidence of schizophrenia [13].

Clinical Presentation

Schizophrenia is characterised by both ‘positive’ and ‘negative’ symptoms, which vary in severity. ‘Positive’ symptoms include auditory hallucinations and disrupted speech, whilst ‘negative symptoms’ include alogia (poverty of speech), anhedonia (loss of interest/enjoyment), social withdrawal, blunted affect, and self-neglect [14].

Despite being a cardinal symptom, not all patients with psychosis will go on to receive a diagnosis of schizophrenia [15]. Rates of remission among individuals with first-episode psychosis can be as high as 60%, with recovery rates of up to 40% [16].

Diagnostic Criteria

The International Classification of Diseases, Tenth Revision (ICD-10) criteria for schizophrenia requires at least one first-rank psychotic symptom or at least two other symptoms. First-rank symptoms include thought insertion, withdrawal, broadcast, bizarre or persistent delusions, or hallucinations (in any modality, but most commonly auditory in the third person). Other symptoms include catatonia, negative symptoms (apathy, blunted affect), or breaks in the train of thought [17].

Basis of disease

Molecular Basis

The molecular mechanism of schizophrenia is likely dependent upon the balance of several neurotransmitters, most importantly, dopamine [18]. Dopamine is a neurotransmitter that is the most abundant catecholamine in the brain [19]. There are four different axonal pathways from which dopaminergic axons project, including nigrostriatal, mesolimbic, mesocortical, and tuberoinfundibular [20] The ‘dopamine hypothesis’ proposes that hyperactivity resulting from excess stimulation of striatal dopaminergic D2 receptors leads to the presence of psychotic, or the ‘positive’ symptoms of schizophrenia [21].

This was initially demonstrated by ‘first-generation’ antipsychotic treatment, such as chlorpromazine, which has been shown to have an inhibitory effect on striatal D2 receptors (a dopamine receptor antagonist) [22]. The dopamine D2 receptor is a G-protein coupled receptor. This is corroborated by PET imaging studies, which have demonstrated typical doses of dopamine receptor antagonists display D2 receptor occupancies above 80% [23].

Whilst, for negative symptoms, it is hypothesised that hypofunction of dopamine in the pre-frontal cortex and other subcortical structures results in apathy, blunt affect, poverty of speech, and cognitive symptoms. This is due to a potential deficit in dopamine transmission at D1 receptors [23]. It is the relationship between these two components that is crucial in the molecular basis of schizophrenia [24]. Nevertheless, recent literature has suggested that the pathophysiology involves dysfunction among serotonergic, glutamatergic, and gamma-aminobutyric acid (GABA) neurotransmission [25].

More recent literature has identified the glutamate synapse as a potential therapeutic target in schizophrenia [26]. Glutamate is the primary excitatory neurotransmitter within the central nervous system. It binds to the N-methyl-D-aspartate (NMDA) receptor, which is fundamental to the glutamate hypothesis. It is proposed that decreased NMDA receptor signalling results in the ‘negative’ symptoms of schizophrenia. This is inferred from the finding that NMDA receptor antagonists such as ketamine produced both negative and positive symptoms in healthy individuals, which ‘resembled’ schizophrenia [27].

There is further evidence that increased glutamate is secondary to GABAergic dysfunction. GABA is the most common inhibitory neurotransmitter [28]. Various post-mortem studies of patients with schizophrenia have demonstrated lower levels of GABA, and/or its marker glutamic acid decarboxylase, which is synthesised from the decarboxylation of glutamate within the midbrain [29-31]. A recent study by Purves-Tyson et al. found pronounced reductions in GABAergic markers, specifically vesicular GABA transporter (VGAT), parvalbumin (PV), GABRA1, GABRA2, and GABRA5 mRNAs within the ventral midbrain in schizophrenia compared to controls. However, they found that changes in these markers were not exacerbated by a higher inflammatory state nor directly correlated with the usage of antipsychotics [32].

Genetic Basis

Genetic factors contribute majorly, but not exclusively, to the development of schizophrenia. Family history is cited as a risk factor - having a first-degree relative with schizophrenia is associated with an odds ratio of 9.8 (95% CI: 6.2-15.5) based on a meta-analysis of twin studies based in Europe, the United States, and Japan [33]. It is highly polygenic, with around 100 genetic loci containing common alleles of small effect [34]. It is the combined effect of the loci, of which there are single nucleotide polymorphisms (SNPs) of varying population frequencies, which contribute towards risk. Nevertheless, most (83%) of schizophrenia cases are sporadic (no affected first-, second- or third-degree relatives) [35].

In addition, rare copy number variants (CNVs), which are either duplications or deletions in large regions (>50 bp) of the genome, are thought to be associated with schizophrenia [36]. Specifically, deletions at 1q21.1 (GJA8 gene), 15q11.2 (CYPFIP1 gene), and 15q13.3 (CHRNA7 gene) have been found to be significantly associated with schizophrenia according to a large genome-wide association study by Stefansson and colleagues [37]. The CNS deletion 22q11.2 is also significantly associated with schizophrenia. Arioka et al. recently demonstrated through induced pluripotent stem cells that this genetic deletion led to reduced expression of protein kinase R-like endoplasmic reticulum kinase (PERK) within the midbrain. This, therefore, is a potential target for therapeutic options for individuals with this deletion [38].

There is an overlap of common risk variants between schizophrenia and other phenotypes such as bipolar disorder and major depressive disorder. This has made it harder to detect specific risk alleles for schizophrenia [39].

## Review

Current treatments

The first-line management of schizophrenia is to offer a ‘second-generation’ antipsychotic, such as olanzapine, quetiapine, or risperidone [40]. These are ‘atypical’ antipsychotics, with the principal difference between ‘typical’ antipsychotics being affinity for receptors other than D2 receptors. Modern ‘atypical’ antipsychotics possess high levels of 5HT2A (serotonergic) affinity.

The main clinical distinction between these two classes of antipsychotics is the presence of extrapyramidal side effects (EPSEs), which include acute dystonias and Parkinsonian-type symptoms. First-generation or ‘typical’ antipsychotics primarily exert their effects through D2 receptor occupancy, leading to EPSEs. Although second-generation antipsychotics carry a reduced incidence of EPSEs, notable metabolic side effects, such as weight gain, remain [41]. Ultimately, the choice of antipsychotic is a complex decision, which depends on clinician and patient preferences, and subsequent choices such as optimising dose are a ‘trial and error’ process [42].

Up to one-third of patients with schizophrenia fail to respond to standard antipsychotics and are considered to have treatment-resistant schizophrenia (TRS) [43]. This is defined as an inadequate response to at least two antipsychotics at an adequate dose for a reasonable time [44].

Clozapine is the only atypical antipsychotic with proven efficacy in treatment-refractory schizophrenia, with two-thirds of patients with treatment resistance predicted to respond beneficially [45]. Its use is complicated by the presence of serious adverse side effects, the most notable being agranulocytosis, which is a potentially fatal reduction in granulocytes. This is predicted to affect approximately 3% of patients during treatment, and while it cannot be reliably predicted, it can be anticipated through routine blood monitoring [46]. In addition, clozapine has a narrow therapeutic window, and some guidelines recommend complementary pharmacokinetic monitoring to optimise dosing via the assessment of drug levels in plasma [47].

Treatment resistance in schizophrenia

Aetiology

Intrinsic to the phenomenon of treatment resistance is a lack of treatment response [44]. This is thought to arise in three ways. First, pathophysiological changes, e.g., upregulation in D2/D3 receptor levels, are so severe that standard treatment is inadequate [48]. Second is the idea that TRS is neurobiologically distinct from treatment-responsive illness, such as the implication of non-dopaminergic pathways, i.e., glutamatergic pathways [49]. Third is the concept that TRS arises from a combination of both factors. These biological factors are thought to contribute to ‘real’ resistance.

‘Pseudo’ resistance, which is not due to the lack of pharmacodynamic action of the drug, but rather influenced by other pharmacokinetic variables such as gender, ethnicity, or genetic factors, can directly affect antipsychotic metabolism [50]. The CYP450 enzyme system is responsible for the metabolism of most atypical antipsychotics, and CYP450 genetic polymorphisms can directly alter drug pharmacokinetics [51]. In addition, other factors such as poor concordance with medication may result in an apparent poor therapeutic response [52].

Antipsychotic Metabolism

Antipsychotics are highly lipophilic with a low clearance rate due to their extensive distribution throughout the body [53]. They are metabolised primarily through three main metabolic pathways: N-oxidation, N-glucuronidation, and phase 1/phase 2 biotransformation [54]. Nonetheless, there is currently a lack of information on N-oxidation and N-glucuronidation within the literature. The major pathway of elimination is hepatic phase I oxidation, which is catalysed by CYP450 enzymes in the liver. Clinically important CYP enzymes include CYP1A2, CYP2D6, CYP3A4, CYP3A5, CYP2B6, and CYP2C19 [55].

CYP450 Enzyme System

Variation within genes coding for CYP enzymes is linked to the differential metabolism of antipsychotics due to differences in drug half-life, clearance, and plasma concentrations [51]. This means that recommended dosing can result in antipsychotic plasma concentrations that are toxic or subtherapeutic, which can lead to the presence of adverse side effects or reduced efficacy [55]. This can explain the wide variability in response to standard doses of antipsychotics [56].

Inter-individual variability in CYP expression due to SNPs results in a lack of expression or altered function of the protein. The large number of variants (alleles) that exist within each CYP450 gene can result in a range of phenotypes: poor metabolisers (PM), intermediate metabolisers (IM), extensive/normal metabolisers (EM), and ultra-rapid metabolisers (UM) [57]. ‘Poor metabolisers’ are thought to possess inactive alleles, whilst ‘extensive metabolisers’ eliminate antipsychotic compounds at ‘normal’ rates. In contrast, ultra-rapid metabolisers eliminate substrates extremely rapidly due to multiple copies of the gene being present [58].

Several examples of genetic polymorphisms have been cited in the literature. Several antipsychotics are known to be extensively metabolised by CYP1A2 (olanzapine and clozapine). The variant rs762551 (1F* haplotype) is considered an inducible variant, which is associated with treatment non-response requiring higher doses. High inducibility of CYP1A2 has been reported in smokers with this variant [59] A study by Eap et al. found that individuals who possessed the *1F variant were confirmed as ‘ultrarapid’ metabolisers [60].

In addition, poor metabolisers of CYP219 have higher serum levels of antipsychotics when compared to extensive or normal metabolisers [61,62]. Those homozygous for CYP2C19* 2 and *3 alleles are poor metabolisers, whilst those homozygous for the CYP2C19*17 alleles are ultra-rapid metabolisers.

Activity Score System

The highly polymorphic nature of CYP450 enzymes makes inferring metaboliser phenotype challenging. The ‘activity score system’ (AS) was introduced as a quantitative measure of phenotype and facilitates the transition from genotype into phenotype [63]. People with the same metaboliser phenotypes can have different activity scores. Thus, the activity score system can further classify inter-individual variability in drug clearance [64].

The activity score system assigns a numeric value to the individual based on the activity of functional alleles it carries for each CYP gene. Since it was first introduced for CYP2D6 specifically, a numeric system has been developed, which ranges from 0 to >3, and up to five metaboliser phenotypes can be recognised in each enzyme. The premise of the numeric system involves assigning each allele a value of 0 (null allele), 0.5 (decreased activity), or 1 (active allele), and combinations of alleles give rise to six possible AS groups (0, 0.05, 1, 1.5, 2, or >3). It is widely assumed among experts that AS = 0 is a poor metaboliser, AS = 0.5 is an intermediate metaboliser, AS = 1.5 or 2 is a normal/extensive metaboliser, and those with AS > 3 is an ultra-rapid metaboliser [65].

The activity score system does not consider specific non-genetic factors, which may influence metaboliser phenotype. Variability in antipsychotic response is influenced by age, ethnicity, the presence of smoking, or inducers/inhibitors of CYP enzymes. ‘Phenoconversion’ is a phenomenon describing how an individual’s predicted metaboliser phenotype does not reflect their actual metaboliser phenotype. It is thought that considering this would make the activity score system more accurate [66].

A key example of how environmental factors can influence CYP enzyme activity is CYP1A2. Notably, cigarette smoking has been shown to induce CYP1A2 activity, whilst medications such as fluoroquinolones and combined oral contraceptives have been shown to inhibit CYP1A2 enzyme activity [67]. Furthermore, certain genetic polymorphisms, such as CYP1A2*1F, for example, which as mentioned previously confers ‘ultra-rapid’ activity exist at a higher frequency in Caucasians [68]. In addition, heavy caffeine consumption has been shown to induce CYP1A2 activity [69]. With respect to CYP2C19, potent inducers such as rifampicin have been referenced in the literature, whilst selective serotonin reuptake inhibitor medications such as fluvoxamine have been shown to inhibit CYP2C19 activity [70,71].

Current challenges in the field

It’s evident that the ‘trial and error approach’ for prescribing antipsychotics is no longer applicable to schizophrenia management, and we need to consider treatment response on a more individual basis [42]. Establishing predictors of antipsychotic response by considering pharmacogenetic factors could enable prescribing and subsequent dosing to optimise treatment. This would make treatment as evidence-based as possible. Nonetheless, the underlying premise that links atypical metabolism to efficacy or side effects remains undisputed.

Phenotyping and genotyping could be clinically useful in predicting treatment response, and thus optimising antipsychotic therapeutics through dosing guidance [72]. Nevertheless, evidence-based guidelines for pharmacogenomics remain scarce, and much of the literature has yet to explore its utility in predicting clinical effects.

There are currently few prospective studies on the role of pharmacogenomics within psychiatry. Skokou et al. carried out a multi-centre prospective study on pre-emptive genome-guided treatment in 1076 people with schizophrenia, depression, and bipolar. This demonstrated that patients with an actionable phenotype had 34.1% fewer adverse drug reactions compared to those in the control group [73].

CPIC, otherwise known as the ‘Clinical Pharmacogenetics Implementation Consortium’, is a project between PharmGKB and the PGRN (Pharmacogenomics Global Research Network) [74,75]. It is responsible for producing clinical guidelines that facilitate the conversion of pharmacogenomic test results into clinically actionable prescribing recommendations for medications [76]. There are currently 26 available CPIC clinical guidelines, none of which are yet specific for antipsychotics.

## Conclusions

The definition of ‘treatment resistance’ is multi-faceted, and patients who are potentially labelled as ‘treatment resistant’ and subsequently placed on clozapine might have ‘pseudo-resistance’. These patients may simply be more likely to end up on clozapine as they are harder to dose properly with other atypical antipsychotics, not because they are ‘biologically resistant’.

Consequently, CYP450 genotype testing could improve therapeutic efficacy in the use of atypical antipsychotics and permit dosage optimisation. Poor metabolisers are notably at a higher risk of adverse side effects due to higher serum antipsychotics whilst ultrarapid metabolisers are hypothesised to be more likely to fail therapy due to decreased exposure to an antipsychotic. Thus, we could potentially reduce the number of patients prescribed clozapine if we utilised pharmacogenomic testing. In addition, although many allelic variants have been observed within the literature for phenotypic metaboliser status, and their genetic variants have been extensively described, the role of some variants is uncertain. Despite many variants being extensively investigated in vitro, in vivo validation remains the gold standard, and until this is possible, the determination of allelic effect may be uncertain.
